# Studying the Ability of Thymol to Improve Fungicidal Effects of Tebuconazole and Difenoconazole Against Some Plant Pathogenic Fungi in Seed or Foliar Treatments

**DOI:** 10.3389/fmicb.2021.629429

**Published:** 2021-02-25

**Authors:** Larisa Shcherbakova, Oleg Mikityuk, Lenara Arslanova, Alexander Stakheev, Denis Erokhin, Sergey Zavriev, Vitaly Dzhavakhiya

**Affiliations:** ^1^Laboratory of Physiological Plant Pathology, All-Russian Research Institute of Phytopathology, Moscow, Russia; ^2^Department of Molecular Biology, All-Russian Research Institute of Phytopathology, Moscow, Russia; ^3^Laboratory of Molecular Diagnostics, Shemyakin-Ovchinnikov Institute of Bioorganic Chemistry, Moscow, Russia

**Keywords:** thymol, chemosensitization to agricultural fungicides, triazoles, *Fusarium* spp., *Bipolaris sorokiniana*, *Parastagonospora nodorum*

## Abstract

Thymol, a secondary plant metabolite possessing antifungal and chemosensitizing activities, disrupts cell wall or membrane integrity and interferes with ergosterol biosynthesis. Thymol also functions as a redox-active compound inducing generation of reactive oxygen species and lipid peroxidation in fungal cells. Previously, we showed thymol significantly enhanced the *in vitro* growth inhibitory effect of difenoconazole against *Bipolaris sorokiniana* and *Parastagonospora nodorum*. More recently, we demonstrated a possibility to use thymol to overcome the resistance of a *P. nodorum* strain able to grow on difenoconazole-containing media. However, potential for thymol to serve as a chemosensitizing agent in seed or plant treatments, to provide an effective suppression of the above-mentioned plant pathogens by triazole fungicides applied in lowered dosages, had yet to be tested. In the work presented here, we showed combined treatments of naturally infected barley seeds with thymol and difenoconazole (Dividend^®^ 030 FS) synergistically exacerbated the protective effect against common root rot agent, *B. sorokiniana*, and other fungi (*Fusarium* spp. and *Alternaria* spp.). Similarly, co-applied treatment of wheat seeds, artificially inoculated with *Fusarium culmorum*, resulted in equivalent reduction of disease incidence on barley seedlings as application of Dividend^®^, alone, at a ten-fold higher dosage. In foliar treatments of wheat seedlings, thymol combined with Folicur^®^ 250 EC (a.i. tebuconazole) enhanced sensitivity of *P. nodorum*, a glume/leaf blotch pathogen, to the fungicide and provided a significant mitigation of disease severity on treated seedlings, compared to controls, without increasing Folicur^®^ dosages. Folicur^®^ co-applied with thymol was also significantly more effective against a strain of *P. nodorum* tolerant to Folicur^®^ alone. No additional deoxynivalenol or zearalenone production was found when a toxigenic *F. culmorum* was cultured in a nutrient medium containing thymol at a concentration used for chemosensitization of root rot agents. Accordingly, *F. culmorum* exposure to thymol at the sensitizing concentration did not up-regulate key genes associated with the biosynthesis of trichothecene or polyketide mycotoxins in this pathogen. Further studies using field trials are necessary to determine if thymol-triazole co-applications result in sensitization of seed- and foliar-associated plant pathogenic fungi, and if thymol affects production of fusarial toxins under field conditions.

## Introduction

Redox-active secondary metabolites of various plants attract continuing interest with regard to the diversity of their biological activity (Jacob, 2014; de Oliveira et al., 2018). Since many of these natural compounds, including thymol, possess antibacterial, antifungal, anti-insect, and other protective properties, they are used in medicine and agriculture or are continually studied for further usage in these areas (Marchese et al., 2016). For example, thymol and thymol-containing essential oils are applied as antimicrobials (Ahmad et al., 2011; Najafloo et al., 2020) and other medical preparations (Riella et al., 2012; El-Marasy et al., 2020) or are proposed as putative ecologically friendly plant-protecting fungicides (Navarro et al., 2011; González-Aguilar et al., 2013; Castillo et al., 2014) and insecticides (Park et al., 2017), as alternatives to chemical pesticides.

An important specific feature of a number of redox-active compounds rendering them as promising agents for control of various pathogenic microorganisms, including some plant pathogenic fungi, is their ability to induce oxidative stress in pathogens or impair fungal antioxidation systems (Kim et al., 2012; Teixeira et al., 2013; Zhang et al., 2015; Shen et al., 2016; Ait Dra et al., 2017; Ji et al., 2018; Ma et al., 2019). In addition, such compounds were shown to augment the effect of antibacterial (Ait Dra et al., 2017; Kissels et al., 2017; Veras et al., 2017; Porter and Monu, 2019; Aksoy et al., 2020) and antifungal (Kordali et al., 2008; Kim et al., 2014, 2015, 2019; Rosato et al., 2018; Schlemmer et al., 2019) agents and to considerably enhance the pathogen sensitivity to antibiotics and antimycotics. In agriculture, this ability of natural redox-active products to improve the efficacy of chemical plant protection (Kim et al., 2017; Shcherbakova et al., 2019) and combat fungal strains resistant to industrial fungicides without increasing dosages (Campbell et al., 2012; Kim et al., 2012) could have significant importance, economically and ecologically.

The damaging and regulatory effects of redox-active molecules are realized through their interaction with lipids, proteins and DNA. These molecules cause lipid peroxidation, oxidative injury of the cell plasma membrane, protein, and DNA oxidation, as well as nitrosylation and S-glutathionylation of proteins (Belozerskaya and Gessler, 2007; Oktyabrsky and Smirnova, 2007). Thymol (2-isopropyl-5-methylphenol), a monoterpene phenol, is the major component of essential oil of *Thymus vulgaris* found also in other *Thymus* and *Lamiaceae* species. Thymol attacks several cellular and metabolic targets in pathogenic fungi. It disrupts cell wall or membrane integrity and interferes with ergosterol biosynthesis (Ahmad et al., 2011; De Lira Mota et al., 2012). Thymol functions also as a redox-active compound, inducing generation of reactive oxygen species and lipid peroxidation in fungal cells (Gao et al., 2016; Shen et al., 2016). Earlier, we showed addition of thymol together with Dividend SC, 3% (a.i. difenoconazole) into nutrient media significantly enhanced the inhibitory effect of this triazole fungicide on the colony growth of *Bipolaris sorokiniana* and *Parastagonospora nodorum* (Dzhavakhiya et al., 2012). Recently, we also demonstrated a potential for using thymol to overcome fungicide resistance of a *P. nodorum* strain that was able to grow on difenoconazole-containing media (Kartashov et al., 2019). However, the possibility to employ thymol as a chemosensitizing agent in seed or plant treatments to provide an effective suppression of the aforementioned plant pathogens by triazole fungicides, applied in lowered dosages, to date, not been tested. At the same time, lowering of effective dosages of these common fungicides could counter expanding pollution of agricultural areas with xenobiotics. In addition, the sensitized resistant strains could be rendered more vulnerable to the fungicides. In this regard, we explored whether seed or foliar treatments with two triazole-based formulations combined with thymol resulted in lowering effective fungicidal dosages in parallel with providing sufficient plant protection and effective control of the aforementioned pathogens. To this end, we studied the protective effect of difenoconazole (Dividend^®^ 030 FS) against *B. sorokiniana*, *F. culmorum* and also accompanying *Fusarium* and *Alternaria* species on seedlings grown from barley and wheat seeds treated with this fungicide in combination with thymol. In another experimental series, protection efficacy of wheat seedlings sprayed with thymol-combined tebuconazole (Folicur^®^ 250 EC) against *P. nodorum* was assessed. We found significant synergistic augmentation of the protective effect of both difenoconazole and tebuconazole when co-applied with thymol. In addition, a possibility to control a tebuconazole-tolerant *P. nodorum* mutant strain by co-application of thymol with Folicur^®^ was showed. We also analyzed deoxynivalenol (DON) and zearalenone (ZEN) production in a toxigenic *F. culmorum* exposed to thymol and profiled expression of key genes associated with the biosynthesis of trichothecene or polyketide mycotoxins in this pathogen. These experiments demonstrated thymol at sensitizing concentrations did not stimulate DON and ZEN biosynthesis. Collectively, our findings confirm the promise of using chemosensitizing agents as an approach to controlling fungal pathogens in agriculture and possibly contributing to development of better environmentally friendly integrated crop protection systems.

## Materials and Methods

### Fungi

Strains of *Fusarium culmorum* (OR-02-37) and *Parastagonospora nodorum* (B-9/47) pathogenic to wheat were provided by the State Collection of Plant Pathogenic Microorganisms at the All-Russian Research Institute of Phytopathology (ARRIP) and the ARRIP Department of Mycology, respectively. *F. culmorum* stock cultures of the fungi maintained on potato dextrose agar slants were resumed by culturing for 10–14 days in Petri plates on the same medium to obtain spore-producing colonies. Suspensions of fungal conidia for inoculations of wheat seeds with *F. culmorum* and detached wheat leaves with *P. nodorum* were prepared according to Shcherbakova et al. (2018) and Shagdarova et al. (2018), respectively.

Samples of barley grain naturally infected with *Bipolaris sorokiniana*, a common root rot agent, were collected from plants grown on the ARRIP experimental plots.

### Thymol and Fungicides

Since thymol (CAS 89-83-8) is slightly soluble in water, solutions of 99% commercial thymol (>99%; REARUS, Russia) in dimethyl sulfoxide, (CAS 67-68-5) 99.9% (Panreac, Spain) were used for seed and leaf treatments. Commercial fungicides tested included Dividend^®^ 030 FS (a.i. difenoconazole, 3%) and Folicur^®^ 250 EC (a.i. tebuconazole, 25%), which are commonly applied for seed and foliar treatments of various crops, including cereals.

### Seed Treatments and Root Rot Development Assay

To select non-phytotoxic dimethyl sulfoxide (DMSO) and thymol concentrations, spring wheat (cv. Zlata) and barley (cv. Zazersky 85) seeds (200 seeds per each treatment) were soaked overnight in aqueous 0.5, 1.0, or 10% DMSO or in thymol dissolved in the DMSO solutions to final concentrations 10, 100, or 1000 ppm. After such treatments seeds were placed on paper towels (filter paper strips of 10 cm × 50 cm, 50 seeds per strip), which were rolled-up and put into beakers with distillated water. Plants were grown at 22°C (day) and 16°C (night), 60% relative humidity and 16-h light period for 7 days. Seeds treated with distillated water were used as controls. The number of germinated seeds, number of seedlings and their length were recorded after 7 days of plant growth.

Prior to conducting sensitization experiments, 50-g samples of non-disinfected barley seeds naturally infected by *B. sorokiniana* and wheat seeds artificially inoculated with *F. culmorum* were treated by a 3-h agitation in a minimal volume of aqueous Dividend^®^ 030 FS allowing complete seed wetting. This was followed by 16–18 h incubation with the fungicide formulation at room temperature, without agitation. Prior to treatment with the fungicide, wheat seeds were disinfected (Shcherbakova et al., 2018), inoculated with *F. culmorum* using 1-h soaking in a conidial suspension (10^5^ conidia/ml) at slow stirring, and then slightly dried. The highest difenoconazole concentration (500 ppm) used for fungicidal treatments of barley and wheat grain samples corresponded to Dividend^®^ 030 FS dose rate recommended for agricultural practice (State Catalogue of Pesticides and Agrochemicals, 2018) for pre-sowing treatments of wheat and barley seeds (2.5 L/1000 kg of grain). To select sub-fungicidal dosages, several lower concentrations of Dividend^®^ ranging from 5.0 to 250 ppm (i.e., from 0.15 to 7.5 ppm based on difenoconazole) were tested. After these treatments, the Dividend-exposed and water-exposed (control) seeds were assayed using rolled-towel test, as described above. After 12–14 days post-inoculation, the number of diseased seedling were counted to evaluate root rot incidence, and disease symptoms on were roots and root necks were visually estimated according to a five-score rating scale (Shcherbakova et al., 2018) to determine the average disease index.

Following the determination of sub-fungicidal Dividend^®^ dosages and non-phytotoxic concentrations of DMSO-dissolved thymol, the respective fungicide and sensitizer dosages were used for combined treatments of barley seeds, naturally infected with *B. sorokiniana*, and wheat seeds, artificially inoculated with *F. culmorum*, as described above. Incidence and disease severity (R%) of common root rot and Fusarium root rot, on barley and wheat seedlings, respectively, were assayed using the rolled-towel test in parallel with the treatments with the fungicide alone. The incidence of *Fusarium* spp. and *Alternaria* spp. accompanying the predominant *B. sorokiniana* infection of barley seeds was also evaluated.

### Evaluation of Folicur^®^ Effect Against *Parastagonospora nodorum*

#### Detached Leaf Assay

In order to determine thymol concentrations, which did not injure wheat leaves but produced a minor disease-suppression effect (Table 1), detached wheat leaves were treated as described previously (Shagdarova et al., 2018). Briefly, *P. nodorum* spores were suspended to a final concentration of 10^6^ conidia/ml in 1% aqueous DMSO or thymol (from 10 to 5000 ppm) dissolved in 1% DMSO. Control conidial suspensions (10^6^ per ml) were prepared in sterile distilled water (SDW). Eight centimeter long leaf fragments were cut from central parts of detached wheat leaves and placed in Petri plates atop of 1% water agar supplemented with benzimidazole (40 μg/ml). Aliquots (10 μl) of the conidial suspensions in DMSO or thymol solutions were dropped on upper (distal) parts of 8-cm leaf fragments. Drops (10-μl) of conidial suspensions (10^6^ conidia/ml) in SDW (control for DMSO-treated detached leaves) or 1% DMSO solutions (control for thymol-treated leaves) were applied to lower (basal) parts of the same 8-cm leaf fragments. *In situ* disease development was recorded on 10 leaf sections per each treatment. An additional plate, in which both distal and basal locations on each of 10 leaf fragment were inoculated with pathogenic conidia in SDW, was prepared to confirm that they was able to cause the disease. Disease symptoms were scored at 5 days post-inoculation according to a five-score rating. Sub-fungicidal dosages of Folicur^®^ 250 EC, tested at concentrations ranging from 0.05 to 0.5 ppm (based on tebuconazole) were determined using the same assay (Table 1). This assay was also used to assess efficacy of Folicur^®^ combined with thymol against a tebuconazole-resistant *P. nodorum* mutant.

**TABLE 1:** Effect of treatments of detached wheat leaves with Folicur^®^ 250 EC or thymol solutions in DMSO on the severity of blotch symptoms caused by *Parastagonospora nodorum*.

Leaf treatments	Average score*	Disease suppression, % of control**
H_2_O	2.97^a^	–
1% DMSO	2.95^a^	0.6
Thymol in 1% DMSO, ppm		
5000	0.55^b^	81.3
500	1.90^c^	35.6
100	2.60^d^	10.5
50	2.80^a^	5.2
10	2.98^a^	0.0
Folicur^®^ in H_2_O, ppm		
0.5	0.40^b^	86.5
0.25	1.63^e^	45.0
0.1	2.13^f^	28.3
0.05	2.85^a^	4.0

*The mean of three replicated assays, 10 detached leaf fragments per treatment in each assay. Mean values indicated with different superscript letters are significant (p ≤ 0.05: t-test for independent variables, STATISTICA 6.0).**Percent suppression calculated relative to scores in corresponding control treatments of leaf fragments: water or 1% DMSO for fungicide or thymol, respectively (see section “Materials and Methods,” “Detached Leaf Assay”). A five score rating scale (1 – small dark lesions; 2 – dark-brown clearly bordered spots, leaf tissue remains green; 3 – light-brown or brown growing spots edged with chlorosis; 4 – light-brown or brown, pycnid formation) was used (Shagdarova et al., 2018).

#### Foliar Treatment of Wheat Seedlings Under Controlled Conditions

Spring wheat seedlings (cv. Khakasskaya) were grown in pots (25–30 seedlings per pot, four pots per treatment) to the two-three leaf stage under controlled conditions (at 21°C during 16-h photoperiod and 18°C during dark, respectively, and 60% relative humidity). Young seedlings were sprayed with *P. nodorum* spore suspensions (10^6^ conidia/ml, 30 ml per each treatment) supplemented with Folicur^®^ alone or DMSO-dissolved thymol alone, each taken at two sub-fungicidal (0.1 and 0.25 ppm of tebuconazole) or two marginally fungitoxic (50 and 100 ppm of thymol) dosages. In parallel, another seedling series was sprayed with fungal conidia suspended in solutions of the fungicide at a concentration of 0.1 ppm combined with thymol at 50 or 100 ppm. To promote infection, pots with treated and inoculated seedlings were maintained in an inocubation chamber at 80–85% relative humidity and 18°C for 24 h. Thereafter plant growing was continued under above-mentioned controlled conditions for 2 weeks. The disease development was scored according to the commonly approved international James’scale (James, 1971) at 14–15 days post-inoculation (the tillering stage Z25, Zadoks et al., 1974) as recommended for registration of early disease symptoms on spring wheat in Non-Chernozem zone of Russia (Sanin and Sanina, 2013).

### Mycotoxin Production Assessment

To estimate production of deoxynivalenol (DON) and zearalenone (ZEN), a toxigenic *F. culmorum* strains, Fc-M-01-55/3, was grown in submerged culture for 7–9 days at 25°Ñ and 220 rpm (New Brunswick^TM^ Excella E25/25R incubation shaker, New Brunswick Scientific, United States) in 250-ml shaker flasks with toxigenesis-promoting liquid Myro medium (Greenhalgh et al., 1988) supplemented with thymol to a final concentration of 50 ppm. Fungal biomass obtained by the submerged culturing was used to assess expression of toxigenesis-associated genes. To prepare other series of samples of fungal mycelia for expression analyses, Fc-M-01-55/3 colonies were also grown in the presence of the same thymol concentration on the same medium with 1.5% agar in 90-mm Petri plates at 25°C for 5–7 days. Thymol was dissolved in 1.0% aqueous DMSO sterilized by filtration through 0.22 μm Millipore membrane filters (MilliporeSigma, United States) and added in the liquid medium or the melted agar medium prior to inoculation. The media were inoculated by addition of a pathogen spore suspension (10^6^ conidia/ml) or by placing a piece of fungal mycelium grown on PDA into the center of the plates. Fungal cultures grown in/on thymol-free media supplemented with corresponding aliquots of 1.0% DMSO under the same conditions served as controls.

#### Mycotoxin Quantification in Submerged *F. culmorum* Cultures

Upon completion of the cultivation, DON and ZEN were quantified by reverse phase HPLC using a Waters 1525 Breeze HPLC system equipped with a Waters 2487 UV detector, applying isolation procedures slightly modified from those described previously (Shcherbakova et al., 2018; Stakheev et al., 2018). Briefly, the submerged culture of each experimental or control flask was extracted twice with an equal volume of ethyl acetate or dichloromethane (to isolate ZEN or DON, respectively) for 1-h on an incubator shaker at 220 rpm and 25°C. Mycelia were separated by centrifugation and dried at 102°C up to a constant weight. Organic phases of supernatants were passed through a layer of anhydrous Na_2_SO_4_ and evaporated on a rotary evaporator at 40°C. Dry residues were dissolved in a mixture of acetonitrile, methanol and water (1:1:0.75) or (1:1:4.0) used as mobile phases in RP-HPLC analyses of ZEN or DON, respectively. Aliquots (10 μL) of the prepared solutions were applied on a temperature-controlled Symmetry C18 (5 μm, 4.6 mm × 150 mm) column followed by toxin elution, using the above-mentioned mobile phases, and detected at 254 nm. Mycotoxin concentrations (μg/mg) were calculated using a calibration curve based on commercial DON and ZEN preparations (Sigma-Aldrich Corp., United States), which were also added in extracts obtained from control fungal cultures as external standards. All samples were analyzed twice. The limits of detection were 0.5 (DON) and 0.2 (ZEN) ng/mg; the recovery levels averaged 81% and 77% for DON and ZEN, respectively.

#### RNA Extraction and Reverse Transcription

Total RNA was extracted from fungal mycelium using RNeasy^®^ Plant Mini Kit (QIAGEN, Germany) according to manufacturer’s protocol. Ten microgram of total RNA were used for cDNA synthesis carried out by the MMLV RT kit (Evrogen JSC, Russia).

#### Quantitative PCR

Expression levels of the following four genes responsible for different steps of trichothecene and polyketide biosynthesis were quantified by qPCR: *TRI5* (trichodiene synthase), *TRI6* (transcription factor), *PKS4* and *PKS13* (polyketide synthesis) were analyzed by qPCR. The translation elongation factor 1 alpha gene (*TEF1*α) served as a control as the most suitable housekeeping gene according to Kim and Yun (2011). The sequences of primers and probes are presented in Table 2. The ClustalX algorithm and Oligo 6.71 program were used for designing gene-specific primers and estimation of their physical properties, respectively. qPCR analyses were performed in a DT-96 thermal cycler (DNA-technology, Russia) according to the following conditions: 94°C for 1 min followed by 45 cycles at 94°C for 10 s, 64°C for 15 s, 72°C for 10 s, and finally 72°C for 3 min. The PCR mix composition was described earlier (Stakheev et al., 2016). Threshold method was used for quantification cycle calculation and analysis of results. qPCR amplification efficiency was calculated using the C_*q*_ slope method. QGene96 software was used for analyzing the qPCR results.

**TABLE 2:** Gene-specific primers and hydrolysis probes used.

Gene	Function	Primers	Probe	Efficiency
*TRI5*	Trichodiene synthase	F: TGGGCACTYGTCAACG R: ATCCARCATCCCTCR4AAAAAG	BHQ1-CCATAGTGCTACGGA(FAMdT)AAGGTTCAATGAGCAG	1.86
*TRI6*	Transcription factor	F: ACTATGAATCACCAACWTTCGAA R: TTGTGTATCCGCCTATAGTGATC	BHQ1- CAAGGGCACCGCAC(FAMdT)GTTGGTTTGTG	1.90
*PKS4*	Polyketide synthase	F: CACATCTCCATCCAAGTTCTG R: GGATCCTGCTTCAAAAAGTGT	BHQ1-GATGGTAGAAGGC(FAMdT)TGTGCATTGTACCGATC	1.91
*PKS13*	Polyketide synthase	F: TGGATGCGACGCCTACAC R: TGCCCGTGTCGGACAATAC	BHQ1- GGCCCAACCTAC(FAMdT)CACTCGACTCGGC	1.95
*TEF1*α	Translation elongation factor 1 alpha	F: GTTGGTCTCATTTTCCTCGATC R: GAGYGGCGGGGTATGAGC	BHQ1- CGACTCGATACGCGCC(FAMdT)GTTACCCCG	1.88

### Data Analysis and Statistics

Data obtained in the experiments involving seed treatments and spraying of wheat seedlings were analyzed using a STATISTICA 6.1 software (StatSoft Inc.). Mean values, standard deviations and standard errors were calculated. Significant difference between treatments and controls were determined using a *t*-test for independent variables at *p* ≤ 0.05. Experiments involving spraying of wheat seedlings (100–120 ones for each treatment) or assessment of *Alternaria* incidence on barley seeds were replicated four times. Other experiments were run in triplicate. Each independent experiment with barley or wheat gain included 200 seeds per treatment.

Protection efficacy (PE) was calculated using formula PE% = Dc – Dt/Dc × 100, where Dc is the average incidence or disease severity in controls, while Dt represents these parameters for treated seeds or seedlings.

To reveal a putative synergy between thymol and tebuconazole or difenoconazole in co-applications on seeds or seedlings, the Limpel criterion, Er ≥ Ee (at *p* ≤ 0.05), was determined (Richer, 1987). In this inequality, Er represented PE% obtained when Dividend^®^ or Folicur^®^ was combined with thymol. Ee is an expected additive effect, i.e., the estimated PE% that could result from summing the protective efficacy of each of components alone calculated using the formula Ee = (X% + Y%) – X% × Y%/100, where X% and Y% are PE% of thymol or the fungicide.

## Results

### Improved Protective Efficacy of Triazoles Combed With Thymol

#### Enhancing the Fungicidal Effect of Dividend^®^ Against Root Rot Agents

The testing phytotoxic effects of DMSO and thymol, which preceded the experiments on individual and thymol-combined fungicidal treatments, showed there was no inhibition of wheat seed germination and seedling formation after seed soaking in 0.5% or 1.0% DMSO. Application of 10% DMSO on barley seeds strongly suppressed germination (Table 3). The highest concentration of thymol (1000 ppm) in 10% DMSO was phytotoxic for wheat and barley based on roll-towel assay. However, thymol at a concentration of 100 ppm dissolved in 1% DMSO was not phytotoxic to seeds of wheat, but was toxic to barley seeds, but did not impede the development of barley seedlings when applied at lower concentrations (Table 3). Based on the results of these initial phytotoxicity assays, thymol at a final concentration of 50 ppm dissolved in 1% DMSO was selected for further chemosensitization experiments on barley and wheat seeds, naturally or artificially infected with fungal root rot agents, respectively.

**TABLE 3:** Influence of wheat and barley seed treatments with dimethyl sulfoxide (DMSO) and thymol dissolved in DMSO on seedling formation.

Plant	Treatments	Seedlings number, % ***
Wheat*	Control, water	96^a^
	10% DMSO	54^b^
	Thymol (1000 ppm) in 10% DMSO	36^c^
	1% DMSO	94^a^
	Thymol (100 ppm) in 1.0% DMSO	92^a^
	Thymol (50 ppm) in 1.0% DMSO	89^a^
	0.5% DMSO	95^a^
	Thymol (10 ppm) in 0.5% DMSO	90^a^
Barley**	Control, water	57^b^
	10% DMSO	9^d^
	Thymol (1000 ppm) in 10% DMSO	0
	1% DMSO	55^c^
	Thymol (100 ppm) in 1.0% DMSO	39^c^
	Thymol (50 ppm) in 1.0% DMSO	55^b^
	Thymol (10 ppm) in 0.5% DMSO	61^b^

*Seeds were surface-disinfect before treatments as described earlier (Shcherbakova et al., 2018). **Non-disinfect seeds naturally infected by fungal root rot agents. ***Percent relative to total seed number. Difference between means (200 seeds per treatment in each of three experiments) indicated with the same letters are insignificant at p ≤ 0.05 (t-test for independent variables, STATISTICA 6.0).

The highest concentration of difenoconazole in our experiments reflected the commercial fungicidal dosage recommended for grain treatments with Dividend^®^ to protect wheat and barley plants against Fusarium and common root rots. For three other treatments, this fungicide was used at dosages half, 10 and a 100 times lower than the highest concentration (i.e., at 7.5, 1.5, and 0.15 ppm based on difenoconazole). These four dosages are indicated below as Dividend/1 (the highest), Dividend/05, Dividend/01, and Dividend/001, respectively. Results of roll-towel assays showed Dividend/1 and Dividend/05 were effective against common root rot infection on barley seedling, while Dividend/01 was the lowest dosage still inhibiting development of *B. sorokiniana*, a predominant seed pathogen, and did not affect seed germination (Table 4). As with naturally infected barley grain, Dividend/01 was the lowest sub-fungicidal dosage when using artificial inoculations of wheat seeds with *F. culmorum* (Table 5). Based on these results, Dividend/01, i.e., the dosage tenfold lower than recommended in agricultural practice, was selected for subsequent chemosensitization experiments.

**TABLE 4:** Development of *B. sorokiniana* on barley seedlings grown from naturally infected seeds treated with Dividend^®^ 030 FS.

Treatments	Seed germination, %	Common root rot (*B. sorokiniana*)*	
		
		Diseased seedlings, %	Average disease index
Control (no fungicide)	87.0^a^	49.4^a^	2.8^a^
Dividend/1	75.0^b^	8.7^b^	0.3^b^
Dividend/05	65.0^b^	20.0^c^	0.8^c^
Dividend/01	87.0^a^	30.5^d^	2.0^d^
Dividend/001	85.0^a^	49.4^a^	2.8^a^

*The means of three experiments (n = 200 per treatment in each). Dividend^®^ 030 FS dosage recommended for grain treatments against Fusarium and common root is indicated as Dividend/1; Dividend/05, /01, and/001 represent twofold-, tenfold-, and hundredfold-diminished dosages, respectively. Means within each column indicated with different superscript letters are significantly different (p ≤ 0.05; t-test for independent variables, STATISTICA 6.0). A five-score rating scale (0 – no disease symptoms; 1– weak symptoms: brown streaks or spots on roots and above; 2 – medium-manifested symptoms; 3 – strong extensive symptoms, 4 – crown and root decaying or plant death) was used (Shcherbakova et al., 2018).

**TABLE 5:** Development of *Fusarium culmorum* on wheat seedlings grown from artificially infected seeds treated with different dosages of Dividend^®^ 030 FS.

Treatments	Seed germination, %	Fusarium root rot (*F. culmorum*)*	
		
		Diseased seedlings, %	Average disease index
Control (no fungicide)	95.0	84.3^a^	3.6^a^
Dividend/1	95.0	54.5^b^	1.9^b^
Dividend/05	94.0	80.3^a^	2.3^b^
Dividend/01	95.0	71.4^c^	3.1^a^
Dividend/001	96.0	79.2^a^	3.5^a^

*Numbers in the columns represent the means of three experiments (n = 200 per treatment in each). Means indicated with different letters are significant at p ≤ 0.05. For additional explanations, see captions to Tables 3, 4.

**FIGURE 1:**
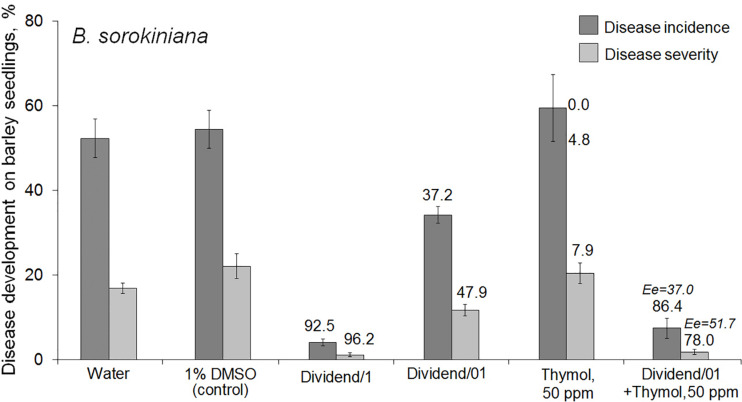
Effect of seed treatments with Dividend^®^ alone, thymol alone or their combination on the development of a root rot agent, *Bipolaris sorokiniana*, on spring barley seedlings (cv. Zazersky 85). Dividend/1, the fungicide dosage recommended for industrial seed treatments. Dividend/01, the fungicide dosage ten times lower than recommended. In treatments involving thymol, it was used as a solution in 1% dimethyl sulfoxide (DMSO). Numbers in regular font above the columns show percent disease suppression observed in experiments (Er). Percent suppression calculated for expected additive effect (Ee) is indicated with italic numbers (see section “Materials and Methods,” “Data Analysis and Statistics”). Disease incidence was determined as the average percent of rotted seedlings compared to DMSO-treated controls. Disease severity was calculated by formula: R% = Σ (n × d)/4N × 100, where n – the number of seedlings with the same disease index; d – the disease index according to a score rating scale (from 0 to 4); N – the number of seedlings per treatment; 4 – the maximal score in the scale (Shcherbakova et al., 2018). Results are expressed as the means of three independent roll-towel assays, 200 seeds per treatment in each assay. Y-bars show SE with a 95% confidence interval (*p* ≤ 0.05, *t*-test for independent variables; STATISTICA v. 6.1, StatSoft Inc.).

Studying the thymol ability to potentiate fungicidal effect of Dividend^®^ toward *B. sorokiniana* showed this redox-active compound was able to sensitize the pathogen to difenoconazole in combined treatments of naturally infected barley seeds. Significantly higher disease suppression was found on seedlings grown from seeds treated with the fungicide combined with thymol, compared to seedlings germinated from seeds treated with Dividend/01 alone (Figure 1). After co-application on seeds, an almost fivefold decrease in pathogen incidence was observed on barley seedlings, and disease severity (R%) was six times lower than that on seedlings grown from seeds, which were soaked in Dividend/01 alone. Moreover, in our experiments, combining the fungicide with thymol provided an almost equal antifungal effect as the tenfold higher dosage, Dividend/1, recommended for agricultural practice. The real antifungal effect of the combined treatments (Er) significantly exceeded not only the impact of Dividend/01, taken alone, but also the calculated additive effect (Ee) expected if there was no synergy (Figure 1). Hence, the elevated protective efficacy of the co-applications resulted from a synergy (Richer, 1987) between thymol and difenoconazole.

In addition to the predominant root rot pathogen, *B. sorokiniana*, we found in preliminary mycological analyses that the barley seeds used were naturally infected with *Fusarium* spp. and *Alternaria* spp. as minor infections with the incidence 10.5% and 7% in DMSO-treated control, respectively. Therefore, we examined the effect of the fungicide-and-thymol treatments against these causative agents, as well. Twofold reduction of *Alternaria* incidence was noticed on the seedlings if Dividend/01 was applied on the seeds conjointly with thymol in two of four performed assays (to 2.7 and 3.5%), but no effect was found in two other ones. Compared to controls, the average incidence of Fusarium rot agents on seedlings decreased to 6.4% or 3.2% after seed treatments with Dividend/01, alone, or Dividend/01 + thymol at 50 ppm, respectively.

**FIGURE 2:**
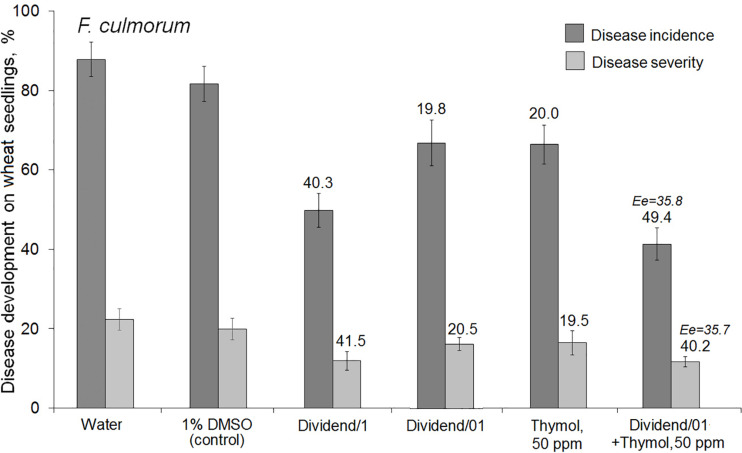
Influence of seed treatments with Dividend^®^ alone, thymol alone or their combination on the development of Fusarium root on spring wheat seedlings (cv. Zlata) from seeds artificially inoculated with *F. culmorum*. Dividend/1 and Dividend/01, the fungicide dosage recommended for industrial seed treatments and tenfold-lower dosage, respectively. Numbers above columns show Er values, while an expected additive effect (Ee) is indicated with italic numbers. The means of three independent roll-towel assays (*n* = 200 seeds per treatment in each assay). Y-bars show SE (*p* ≤ 0.05). For additional explanations, see caption to Figure 1 and section “Materials and Methods,” “Data Analysis and Statistics.”

Co-application of thymol and Dividend^®^ on artificially inoculated wheat seeds also resulted in augmentation of the suppressive activity of this triazole fungicide against *F. culmorum* (Figure 2). After co-applications of Dividend/01 combined with thymol, both incidence and severity of the disease on wheat seedlings were almost twice as low compared to application of Dividend/01 only. Comparisons of Er with Ee values suggested the augmented fungicidal effect obtained in experiments involving artificial seed inoculation was most likely a result of both sensitizing and fungitoxic activity of thymol. Thus, reduction of *F. culmorum* incidence on wheat seedlings after the fungicide-thymol co-application significantly exceeded the estimated additive effect. However, mitigation of disease severity by these combined applications was induced to a certain degree by an additive interaction of the fungicide and thymol rather than by chemosensitization (Figure 2). Nevertheless, joint influence of Dividend/01 and thymol on the Fusarium root rot agent had total fungicidal effect equal to the effect of a tenfold higher dosage of the fungicide, alone.

#### Enhancing the Fungicidal Effect of Folicur^®^ Against *Parastagonospora nodorum*

**FIGURE 3:**
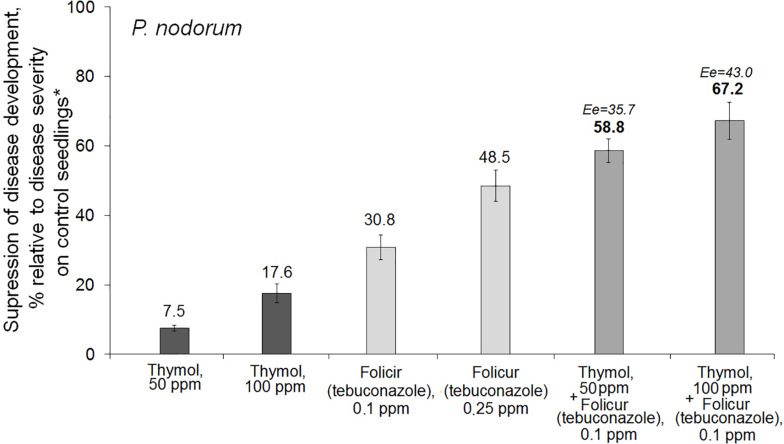
Histograms showing the inhibitory effect of Folicur^®^ EC 250 on development of *Parastagonospora nodorum*, a glume/leaf blotch agent, on wheat seedlings treated with thymol alone and the fungicide either alone or in two combinations with thymol. *The average percentage of disease development (100–120 seedlings per treatment); control plants were sprayed with *P*. *nodorum* conidia in water. The means of four experiments; Y-bars indicate SE (see section “Materials and Methods,” “Data Analysis and Statistics” and caption to Figure 1 for additional explanations).

Thymol was found to augment protective action of Folicur^®^ EC 250 against *P. nodorum* in foliar treatments of wheat seedlings grown under controlled conditions. Spraying of wheat seedlings with thymol at 50 ppm or with the fungicide at 0.1 ppm did not sufficiently suppress disease development. In contrast, the combined treatment with the same doses of tebuconazole and thymol increased the antifungal effect. The co-applications resulted in disease suppression at a level exceeding the effect of the higher dosage of the fungicide (0.25 ppm) after application of fungicide, alone (Figure 3). Calculation of Limpel’s criterion (Richer, 1987) pointed to existence of synergy between thymol and tebuconazole in both combinations used. Er values (58.8 and 67.2%) exceeded Ee values (35.7 and 43.0%) at *p* = 0.02 and 0.03, respectively (Figure 3). Thus, joint treatments of wheat seedlings with fungicide and sensitizer produced an enhanced protective effect without increasing fungicide dosage.

**FIGURE 4:**
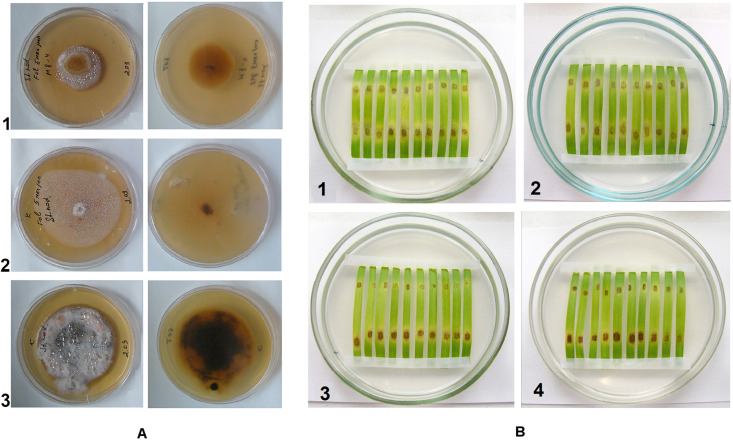
**(A)** Colonies of tebuconazole-tolerant mutant **(1)** and wild **(2, 3)** strains of *Parastagonospora nodorum*, grown on potato-dextrose agar containing Folicur^®^ **(1** and **2)** or not containing Folicur^®^ **(3)** Photos in the right column show fungal colonies on reverse side of the plates. **(B)** *P. nodorum* blotch lesions on leaf fragments cut from detached wheat leaves. **(1, 2, 4)**, tebuconazole-tolerant mutant; **(3)**, wild strain. Upper (distal) portions of leaf fragments are inoculated with a suspension of conidia (10^6^/ml) supplemented with Folicur^®^ alone **(1)**, thymol alone **(2)** or their mixture **(3** and **4)** containing the same dosages as used for **(1, 2)**. Lower (basal) portions of leaf fragments are inoculated with conidia suspended in water to the same final concentration of 10^6^ per ml.

Thymol-Folicur^®^ combinations were used against a *P. nodorum* mutant strain manifesting tolerance to this fungicide. This mutant was able to grow on PDA supplemented with tebuconazole concentrations up to 1.25 ppm (i.e., up to 5 ppm based on the formulation) that was lethal to a sensitive wild strain (Figure 4A). Both thymol and tebuconazole at dosages of 50 ppm and 0.05 ppm, respectively, did not show a protective effect against the tolerant pathogen when applied separately. In contrast, the combined treatment at these dosages resulted in an observable mitigation of lesions on detached leaves inoculated with either the wild or with the tolerant strain (Figure 4B).

### Mycotoxin Production by Toxigenic *Fusarium culmorum* Exposed to Thymol

To ensure thymol did not induce production of sesquiterpenoid or polyketide fusariotoxins, DON and ZEN content was analyzed by HPLC after culturing a toxin-producing *F. culmorum* strain in media supplemented with thymol at 50 ppm, the concentration used to sensitize the pathogen to Dividend^®^. In addition, expression levels of selected genes associated with mycotoxin production by *Fusarium* fungi (Lysøe et al., 2006; Kimura et al., 2007; Villafana et al., 2019) were explored.

**FIGURE 5:**
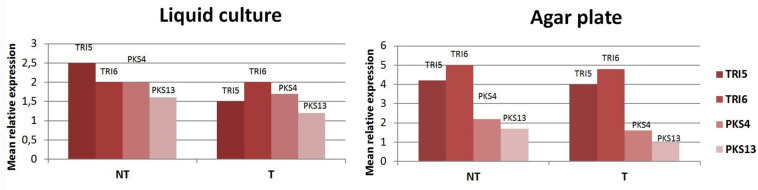
Mean relative expression levels of *TRI5*, *TRI6*, *PKS4*, and *PKS13* after culturing *Fusarium culmorum* in submerged cultures and on agar plates. **T**, *F. culmorum* cultures exposed to thymol at a sensitizing concentration of 50 ppm; **NT**, non-exposed cultures.

No statistically significant increase of DON and ZEN concentrations compared to control was determined using HPLC (Table 6). Expression levels of key mycotoxin biosynthetic genes of *F. culmorum* were analyzed by qPCR for four genes, two (*TRI5* and *TRI6*) localized in the trichothecene biosynthetic cluster and two (*PKS4* and *PKS13*) belonging to the polyketide biosynthetic cluster. Gene expression was analyzed 2 days before the maximal accumulation of DON or ZEN in thymol-containing culture media inoculated with the pathogen. Levels of *TRI5*, *TRI6*, *PKS4*, and *PKS13* expression were compared to those in control fungal cultures grown without thymol under the same conditions. Expression of the control gene (*TEF1*α) was stable in all the samples tested. Expression levels of four target genes in thymol-exposed samples were the same or slightly lower those of the control, in both submerged and agar cultures (Figure 5).

**TABLE 6:** Deoxynivalenol (DON) and zearalenone (ZEN) production by a toxigenic *F. culmorum* strain cultured in thymol-containing Myro medium.

Thymol, ppm	Mycotoxins, μg/mg of dried mycelium weight*			
	DON	p**	ZEN	*p*
0.0	0.16 ± 0.04	0.067	0.98 ± 0.19	0.085
50.0	0.12 ± 0.02	1.08 ± 0.17

*Mean ± SD of three independent cultivation experiments, three replications per treatment in each. **The differences of treatments from the control (Thymol, 0.0 ppm) are insignificant (p > 0.05).

## Discussion

Thymol appears to be a promising chemosensitizing agent for various pathogenic and opportunistic fungi, based on several *in vitro* studies. For instance, Kim et al. (2015) found thymol synergistically interacted with another redox-active molecule, 2-hydroxy-4-methoxybenzaldehyde (2H4M), augmenting the efficacy of cell wall disruption by these compounds and enhancing their effect against *Aspergillus fumigatus* and *Aspergillus parasiticus*. Thymol and 2H4M co-applications resulted also in a lowering of fungicidal concentrations and an elevated antifungal activity of these compounds for *Aspergillus flavus*, *Saccharomyces cerevisiae*, and *Penicillium* strains, despite fractional inhibitory concentration indices (FICIs) pointing to the absence of a calculated synergism (Kim et al., 2015). Accordingly, Guarda et al. (2011) demonstrated the synergistic effect between thymol and its isomer, caracole, toward *Aspergillus niger* growth inhibition. A partial synergism was revealed when thymol was tested in combinations with monoterpene- and phenol-containing citronella and garlic essential oils used to inhibit *Penicillium corylophilum* causing a spoilage of dried meat foods (Ji et al., 2019). Thymol displayed also *in vitro* synergistic interaction with nystatin (FICI = 0.17) used against a zoophilic yeast *Malassezia pachydermatis* (Schlemmer et al., 2019).

Previously, we found thymol synergistically augmented *in vitro* ability of three fungicidal formulations to inhibit the growth of fungal colonies (Dzhavakhiya et al., 2012). In this research, enhanced antifungal activity was shown for Dividend^®^ toward *B. sorokiniana and P. nodorum*, Folicur^®^ against *Alternaria alternata*, and a strobilurin fungicide, Quadris^®^, against *B. sorokiniana*, *Phoma glomerata*, and *P. nodorum*. The current work, reported here, represents a next stage of our investigations involving seed and foliar treatments of barley and wheat with Dividend^®^ or Folicur^®^ combined with thymol to improve suppression of *B. sorokiniana* and *F. culmorum*, or *P. nodorum*, commonly controlled by difenoconazole and/or tebuconazole containing fungicides (State Catalogue of Pesticides and Agrochemicals, 2018). These fungi belong to the most destructive seed-born and leaf-associated pathogens of cereals (Bhathal et al., 2003; Shcherbakova et al., 2018) and cause a number of economically significant diseases, such as wheat and barley common root (*B. sorokiniana*) and Fusarium crown-foot-root rot, head blight (*F. culmorum*), and glume/leaf blotch of wheat (*P. nodorum*).

At certain concentrations, thymol is known to be phytotoxic and was reported to inhibit seed germination and growth of some weed plants (Kordali et al., 2008; Araniti et al., 2020). However, in our study we preliminarily determined DMSO-dissolved thymol concentrations for use in chemosensitization which did not negatively affect seed germination, or cause lesions on wheat leaves, and only marginally prevented pathogen development (Tables 1, 3). In parallel, fungicide dosages of Dividend^®^ and Folicur^®^, selected for our experiments to show sensitization by thymol, were at levels that did not protect seedlings or only provided a minor protective effect, and were far lower than dosages recommended for agricultural use (Tables 1, 4, 5). Results obtained using co-applications of thymol with Dividend^®^ on naturally infected barley grain and artificially inoculated wheat seeds, as well as results of foliar treatments of wheat seedlings with Folicur^®^ combined with thymol, showed this redox-active plant metabolite significantly improved the protective effects of difenoconazole and tebuconazole. In all cases, the combined treatments resulted in significantly more effective suppression of plant diseases than the application of the fungicide alone (Figures 1, 3). Moreover, compositions of tested fungicides and the sensitizer provided the same effective protection as Dividend^®^, alone, or Folicur^®^, alone, if these fungicides were applied at 10 or 2.5 times higher dosages, respectively (Figures 1, 3). The fact that Er values significantly exceeded Ee values showed this efficacy was synergistically enhanced by chemosensitization of the pathogens to both triazoles. The one exception was reduction of disease severity as an additive effect of difenoconazole and thymol against *F. culmorum*. Although in two of four performed assays, co-application of thymol and Dividend^®^ on barley seeds did not enhance the protective effect against Alternaria root rot, a high efficacy of thymol-combined Dividend/01 toward the predominant *B. sorokiniana* provided the same reduction of incidence of common root rot as the fungicide alone at its highest dosage (Dividend/1) in all experiments.

Currently, a general concept explaining augmentation of pathogen sensitivity to antifungal agents co-applied with a sensitizing compound is based on the sensitizing compound and fungicide attacking different pathways of fungal metabolism or different stages of the same metabolic pathway. This concept seems to be quite reasonable to explain a possible mechanism underlying enhanced fungicidal effects demonstrated in our experiments. Indeed, for various natural sensitizers, destabilizing structural integrity of cellular and vacuolar membranes, induction of oxidative stress *via* ROS generation with the added provocation of osmotic stress, are main mechanisms augmenting sensitivity of fungi to triazoles and other antimycotics (Campbell et al., 2012). Although all potential mechanisms of thymol activity against plant pathogenic fungi are, so far, poorly understood, its antimicrobial effect has been reported to be mainly associated with extensive damage of fungal membranes. This effect is due to the thymol hydroxyl group binding to spore and hypha membranes and lipid peroxidation impairing ergosterol biosynthesis in fungal cells (Ahmad et al., 2011; De Lira Mota et al., 2012; Gao et al., 2016). Like other redox-active compounds with sensitizing activity, thymol causes oxidative and osmotic stress responses. For instance, it induces ROS, and then NO generation in *A. flavus* resulting in lysis and death of fungal spores (Shen et al., 2016). It is also hypothesized that, in some plant pathogens, MAPK signaling associated with reactions to the above-mentioned stresses (Campbell et al., 2012) can be modulated by thymol (Gao et al., 2016). Simultaneously, thymol and triazoles affect different targets. In contrast to thymol, difenoconazole and tebuconazole inhibit 14α-demethylase (CYP51) required for cytochrome P450-dependent oxidative demethylation of 24-methylene-24,25-dihydrolanosterol, a precursor of ergosterol (FRAC, 2020). Such multiple targeting of fungal metabolism can significantly weaken plant pathogens, enhancing their sensitivity to thymol and fungicidal doses ineffective alone, and thus elevate the suppressive effect of triazoles.

It is possible that mitigation of disease symptoms on detached wheat leaves inoculated with a tebuconazole-tolerant *P. nodorum* mutant was provided by synergy between the fungicide and thymol. Results obtained using detached leaf assays showed thymol can be co-applied with Folicur^®^ to suppress development of a resistant pathogen in plant tissues at the same fungicidal dose inhibiting a sensitive wild strain. These findings warrant further investigations on sensitization of sensitive and resistant *P. nodorum* strains under expanded assay conditions, such as greenhouse or field experiments.

Interestingly, in this study, thymol concentration enhancing the sensitivity of both *B. sorokiniana* and *P. nodorum* in seed and seedling treatments, respectively, exceeded *in vitro* sensitizing dose selected in prior experiments (Dzhavakhiya et al., 2012) using dilutions based on checkerboard assays (Odds, 2003). Hence, vegetation experiments are necessary as the first step in validating if sensitization discovered *in vitro* reflects augmentation of antifungal effects on plants in the field.

Some redox-active compounds including thymol, which induce oxidative stress, were reported to activate the biosynthesis of polyketide mycotoxins in *Aspergillus* spp. (Kim et al., 2015; Roze et al., 2015; Dzhavakhiya et al., 2016) and trichothecene production in some *Fusarium* species (Gardiner et al., 2010). Taking this into account, we cultured a DON- and ZEA-producing *F. culmorum* strain in the presence of thymol at 50 ppm, the same concentration used for seed treatments to improve the effect of lowered Dividend dosages. In these experiments, we evaluated production levels of both mycotoxins in the cultural liquor and expression of genes participating in mycotoxigenesis. Results showed that treatment of fungal cultures by thymol did not boost DON- and ZEA production, and did not up-regulate key toxin biosynthetic genes (Table 6 and Figure 5). In the case of gene TRI5, encoding trichodiene synthase, and genes *PKS4* and *PKS13*, encoding polyketide synthases, the expression levels were slightly lower after thymol treatment. Expression levels of *TRI6*, a gene encoding a global transcription regulator, were the same in both treated and non-treated cultures. These results suggest thymol, at the sensitizing concentration, does not potentiate expression of genes associated with the biosynthesis of the test fusarial toxins and their subsequent production.

In conclusion, this research showed promise for applying thymol to improve fungicidal efficacy of difenoconazole and tebuconazole against cereal root rot and glume/leaf blotch agents, including fungicide-resistant mutants. Importantly, the same concentration of this sensitizer may be used in both seed and foliar treatments against different cereal pathogens. However, further research is necessary to confirm if the thymol-triazole co-applications will result in sensitization of seed- and leaf-associated plant pathogenic fungi or regulation of mycotoxin production under field conditions. Additional investigations are also needed to determine if thymol affects expression of other toxigenesis-related genes in other Fusarium root rot agents.

## Data Availability Statement

The primers and fluorogenic probes were designed based on the alignment of the following sequences retrieved from GenBank (https://www.ncbi.nlm.nih.gov/nucleotide/): U22464, AY130290, MN313473-313507 (TRI5); MH514940-514957, MH001614-MH001648 (TRI5 and TRI6); AY429625, XM009259983 (PKS4), AY495638, JF966273 (PKS13); DQ019316 (PKS4 and PKS13); KY205745-205748, DQ382164-382166 (TEF1a).

## Author Contributions

LS and VD conceived the concept of the research and designed the experiments. OM, LA, and DE performed the main experiments, prepared the reagents, materials, and analysis tools. AS performed the experiments on expression of genes associated with mycotoxigenesis. LS and AS wrote the original draft and prepared the figures and tables. LS, VD, and SZ reviewed and edited the drafts of the manuscript. All authors reviewed and approved the manuscript.

## Conflict of Interest

The authors declare that the research was conducted in the absence of any commercial or financial relationships that could be construed as a potential conflict of interest.

## References

[B1] Ahmad A. Khan A. Akhtar F. Yousuf S. Xess I. Khan L. A. (2011). Fungicidal activity of thymol and carvacrol by disrupting ergosterol biosynthesis and membrane integrity against *Candida*. *Eur. J. Clin. Microbiol. Infect. Dis.* 30 41–50. 10.1007/s10096-010-1050-8 20835742

[B2] Ait Dra L. Brahim M. Boualy B. Aghraz A. Barakate M. Oubaassine S. (2017). Chemical composition, antioxidant and evidence antimicrobial synergistic effects of *Periploca laevigata* essential oil with conventional antibiotics. *Ind. Crops Prod.* 109 746–752. 10.1016/j.indcrop.2017.09.028

[B3] Aksoy C. S. Avci F. G. Ugurel O. M. Atas B. Sayar N. A. Akbulut B. S. (2020). Potentiating the activity of berberine for *Staphylococcus aureus* in a combinatorial treatment with thymol. *Microb. Pathog.* 149:104542. 10.1016/j.micpath.2020.104542 33010366

[B4] Araniti F. Miras-Moreno B. Lucini L. Landi M. Abenavoli M. R. (2020). Metabolomic, proteomic and physiological insights into the potential mode of action of thymol, a phytotoxic natural monoterpenoid phenol. *Plant Physiol. Biochem.* 153 141–153. 10.1016/j.plaphy.2020.05.008 32502716

[B5] Belozerskaya T. A. Gessler N. N. (2007). Reactive oxygen species and the strategy of antioxidant defense in fungi: a review. *Appl. Biochem. Microbiol.* 43 506–515. 10.1134/S0003683807050031 18038677

[B6] Bhathal J. Loughman R. Speijers J. (2003). Yield reduction in wheat in relation to leaf disease from yellow (tan) spot and Septoria nodorum blotch. *Eur. J. Plant Pathol.* 109 435–443. 10.1023/A:1024277420773

[B7] Campbell B. C. Chan K. L. Kim J. H. (2012). Chemosensitization as a means to augment commercial antifungal agents. *Front. Microbiol.* 3:79. 10.3389/fmicb.2012.00079 22393330 PMC3289909

[B8] Castillo S. Pérez-Alfonso C. O. Martínez-Romero D. Guillén F. Serrano M. Valero D. (2014). The essential oils thymol and carvacrol applied in the packing lines avoid lemon spoilage and maintain quality during storage. *Food Control* 35 132–136. 10.1016/j.foodcont.2013.06.052

[B9] De Lira Mota K. S. de Oliveira Pereira F. de Oliveira W. A. Lima I. O. de Oliveira Lima E. (2012). Antifungal activity of *Thymus vulgaris* L. essential oil and its constituent phytochemicals against *Rhizopus oryzae*: interaction with ergosterol. *Molecules* 17 14418–14433. 10.3390/molecules171214418 23519243 PMC6268362

[B10] de Oliveira M. S. Almeida M. M. Salazar M. de L. A. R. Pires F. C. S. Bezerra F. W. F. (2018). “Potential of medicinal use of essential oils from aromatic plants,” in *Potential of Essential Oils*, ed. El-Shemy H. (London: InTech), 1–21.

[B11] Dzhavakhiya V. Shcherbakova L. Semina Y. Zhemchuzhina N. Campbell B. (2012). Chemosensitization of plant pathogenic fungi to agricultural fungicides. *Front. Microbiol.* 3:87. 10.3389/fmicb.2012.00087 22408641 PMC3297821

[B12] Dzhavakhiya V. G. Voinova T. M. Popletaeva S. B. Statsyuk N. V. Limantseva L. A. Shcherbakova L. A. (2016). Effect of various compounds blocking the colony pigmentation on the aflatoxin B1 production by *Aspergillus flavus*. *Toxins* 8:11. 10.1006/EXCR.2002.5662 27801823 PMC5127110

[B13] El-Marasy S. A. El Awdan S. A. Hassan A. Abdallah H. M. I. (2020). Cardioprotective effect of thymol against adrenaline-induced myocardial injury in rats. *Heliyon* 6:e04431. 10.1016/j.heliyon.2020.e04431 32715125 PMC7378581

[B14] FRAC (2020). *FRAC Code List* ©. Available online at: https://www.frac.info/docs/default-source/publications/frac-code-list/frac-code-list-2020-finalb16c2 b2c512362eb9a1eff00004acf5d.pdf?sfvrsn=54f499a_2 (accessed January 17, 2021).

[B15] Gao T. Zhou H. Zhou W. Hu L. Chen J. Shi Z. (2016). The fungicidal activity of thymol against *Fusarium graminearum* via inducing lipid peroxidation and disrupting ergosterol biosynthesis. *Molecules* 21:770. 10.3390/molecules21060770 27322238 PMC6272974

[B16] Gardiner D. M. Kazan K. Praud S. Torney F. J. Rusu A. Manners J. M. (2010). Early activation of wheat polyamine biosynthesis during Fusarium head blight implicates putrescine as an inducer of trichothecene mycotoxin production. *BMC Plant Biol.* 10:298–302. 10.1186/1471-2229-10-289 21192794 PMC3022911

[B17] González-Aguilar G. Ansorena M. Viacava G. Roura S. Ayala-Zavala J. (2013). “Plant essential oils as antifungal treatments on the postharvest of fruit and vegetables,” in *Antifungal Metabolites from Plants*, eds Razzaghi-Abyaneh M. Rai M. (Berlin: Springer), 429–446.

[B18] Greenhalgh R. Blackwell B. A. Savard M. Miller J. D. Taylor A. (1988). Secondary metabolites produced by *Fusarium sporotrichioides* DAOM165006 in liquid culture. *Agric. Food Chem.* 36 216–219. 10.1021/jf00079a054

[B19] Guarda A. Rubilar J. F. Miltz J. Galotto M. J. (2011). The antimicrobial activity of microencapsulated thymol and carvacrol. *Int. J. Food Microbiol.* 146 144–150. 10.1016/j.ijfoodmicro.2011.02.011 21411168

[B20] Jacob C. (2014). Redox active natural products and their interaction with cellular signaling pathways. *Molecules* 19 19588–19593. 10.3390/molecules191219588 25432010 PMC6271017

[B21] James W. S. (1971). An illustrated series of assessment keys for plant diseases, their preparation and usage. *Canadian Plant Dis. Survey* 51 36–65.

[B22] Ji D. Chen T. Ma D. Liu J. Xu Y. Tian S. (2018). Inhibitory effects of methyl thujate on mycelial growth of *Botrytis cinerea* and possible mechanisms. *Postharvest Biol. Technol.* 142 46–54. 10.1016/j.postharvbio.2018.04.003

[B23] Ji H. Kim H. Beuchat L. R. Ryu J. H. (2019). Synergistic antimicrobial activities of essential oil vapours against *Penicillium corylophilum* on a laboratory medium and beef jerky. *Int. J. Food Microbiol.* 291 104–110. 10.1016/j.ijfoodmicro.2018.11.023 30481661

[B24] Kartashov M. I. Shcherbakova L. A. Statsyuk N. V. Dzhavakhiya V. G. (2019). Co-application of difenoconazole with thymol results in suppression of a *Parastagonospora nodorum* mutant strain resistant to this triazole. *KnE Life Sci.* 4 1097–1106. 10.18502/kls.v4i14.570

[B25] Kim H. K. Yun S. H. (2011). Evaluation of potential reference genes for quantitative RT-PCR analysis in *Fusarium graminearum* under different culture conditions. *Plant Pathol. J.* 27 301–309. 10.5423/PPJ.2011.27.4.301

[B26] Kim J. Chan K. L. Cheng L. W. Tell L. A. Byrne B. A. Clothier K. (2019). High efficiency drug repurposing design for new antifungal agents. *Methods Protoc.* 2:31. 10.3390/mps2020031 31164611 PMC6632159

[B27] Kim J. H. Chan K. L. Faria N. G. Campbell B. C. (2012). Targeting the oxidative stress response system of fungi with safe, redox-potent chemosensitizing agents. *Front. Microbiol.* 3:88. 10.3389/fmicb.2012.00088 22438852 PMC3305922

[B28] Kim J. H. Chan K. L. Mahoney N. E. (2015). Augmenting the activity of monoterpenoid phenols against fungal pathogens using 2-hydroxy-4-methoxybenzaldehyde that target cell wall integrity. *Int. J. Mol. Sci.* 16 26850–26870. 10.3390/ijms161125988 26569223 PMC4661847

[B29] Kim J. H. Mahoney N. E. Chan K. L. Campbell B. C. Haff R. P. Stanker L. H. (2014). Use of benzoanologs to enhance antimycotic activity of kresoxim methyl for control of aflatoxigenic fungal pathogens. *Front. Microbiol*. 5:87. 10.3389/fmicb.2014.00087 24639673 PMC3945611

[B30] Kim K. Lee Y. Ha A. Kim J. I. Park A. R. Yu N. H. (2017). Chemosensitization of *Fusarium graminearum* to chemical fungicides using cyclic lipopeptides produced by *Bacillus amyloliquefaciens* strain JCK-12. *Front Plant Sci.* 8:2010. 10.3389/fpls.2017.02010 29230232 PMC5711811

[B31] Kimura M. Tokai T. Takahashi-Ando N. Ohsato S. Fujimura M. (2007). Molecular and genetic studies of *Fusarium* trichothecene biosynthesis: pathways, genes, and regulation. *Biosci. Biotechnol. Biochem.* 71 2105–2123. 10.1271/bbb.70183 17827683

[B32] Kissels W. Wu X. Santos R. R. (2017). Short communication: interaction of the isomers carvacrol and thymol with the antibiotics doxycycline and tilmicosin: in vitro effects against pathogenic bacteria commonly found in the respiratory tract of calves. *J. Dairy Sci.* 100 970–974. 10.3168/jds.2016-11536 28012625

[B33] Kordali S. Cakir A. Ozer H. Cakmakci R. Kesdek M. Mete E. (2008). Antifungal, phytotoxic and insecticidal properties of essential oil isolated from Turkish *Origanum acutidens* and its three components, carvacrol, thymol and p-cymene. *Bioresour. Technol.* 99 8788–8795. 10.1016/j.biortech.2008.04.048 18513954

[B34] Lysøe E. Klemsdal S. S. Bone K. R. Frandsen R. J. N. Johansen T. Thrane U. (2006). The PKS4 gene of *Fusarium graminearum* is essential for zearalenone production. *Appl. Environ. Microbiol.* 72 3924-3932. 10.1128/AEM.00963-05 16751498 PMC1489647

[B35] Ma D. Cui X. Zhang Z. Li B. Xu Y. Tian S. (2019). Honokiol suppresses mycelial growth and reduces virulence of *Botrytis cinerea* by inducing autophagic activities and apoptosis. *Food Microbiol.* 88:103411. 10.1016/j.fm.2019.103411 31997759

[B36] Marchese A. Orhan I. E. Daglia M. Barbieri R. Di Lorenzo A. Nabavi S. F. (2016). Antibacterial and antifungal activities of thymol: a brief review of the literature. *Food Chem.* 210 402–414. 10.1016/j.foodchem.2016.04.111 27211664

[B37] Najafloo R. Behyari M. Imani R. Nour S. (2020). A mini-review of thymol incorporated materials: applications in antibacterial wound dressing. *J. Drug Deliv. Sci. Technol.* 60:101904. 10.1016/j.jddst.2020.101904

[B38] Navarro D. Diaz-Mula H. M. Guillen F. Zapata P. J. Castillo S. Serrano M. (2011). Reduction of nectarine decay caused by *Rhizopus stolonifer*, *Botrytis cinerea* and *Penicillium digitatum* with Aloe vera gel alone or with the addition of thymol. *Int. J. Food Microbiol.* 151 241–246. 10.1016/j.ijfoodmicro.2011.09.009 21974979

[B39] Odds F. C. (2003). Synergy, antagonism, and what the chequerboard puts between them. *J. Antimicrob. Chemother.* 52:1. 10.1093/jac/dkg301 12805255

[B40] Oktyabrsky O. N. Smirnova G. V. (2007). Redox regulation of cellular functions. *Biochemistry (Moscow)* 72 132–145. 10.1134/S0006297907020022 17367290

[B41] Park J.-H. Jeon Y.-J. Lee C.-H. Chung N. Lee H.-S. (2017). Insecticidal toxicities of carvacrol and thymol derived from *Thymus vulgaris* Lin. against *Pochazia shantungensis* Chou & Lu., newly recorded pest. *Sci. Rep.* 7:40902. 10.1038/srep40902 PMC5247674 28106093

[B42] Porter J. A. Monu E. A. (2019). Evaluating the antimicrobial efficacy of white mustard essential oil alone and in combination with thymol and carvacrol against *Salmonella*. *J. Food Prot*. 82 2038–2043. 10.4315/0362-028X.JFP-19-029 31692393

[B43] Richer D. L. (1987). Synergism: a patent view. *Pest Manag. Sci.* 19 309–315. 10.1002/ps.2780190408

[B44] Riella K. R. Marinho R. R. Santos J. S. Pereira-Filho R. N. Cardoso J. C. Albuquerque-Junior R. L. C. (2012). Anti-inflammatory and cicatrizing activities of thymol, a monoterpene of the essential oil from *Lippia gracilis*, in rodents. *J. Ethnopharmacol.* 143 656–663. 10.1016/j.jep.2012.07.028 22885071

[B45] Rosato A. Carocci A. Catalano A. Clodoveo M. L. Franchini C. Corbo F. (2018). Elucidation of the synergistic action of *Mentha piperita* essential oil with common antimicrobials. *PLoS One*. 13:e0200902. 10.1371/journal.pone.0200902 30067803 PMC6070247

[B46] Roze L. V. Laivenieks M. Hong S. Y. Wee J. Wong S. S. Vanos B. (2015). Aflatoxin biosynthesis is a novel source of reactive oxygen species – a potential redox signal to initiate resistance to oxidative stress? *Toxins.* 7 1411–1430. 10.3390/toxins7051411 25928133 PMC4448155

[B47] Sanin S. S. Sanina A. A. (2013). *Septoria Leaf Blotch and Stagonospora Leaf/Glume Blotch of Wheat: Diagnostics, Phytosanitary Observations, and Plant Protection Managing.* Moscow: AMA Press.

[B48] Schlemmer K. Jesus F. P. Tondolo J. S. Weiblen C. Azevedo M. Machado V. (2019). In vitro activity of carvacrol, cinnamaldehyde and thymol combined with antifungals against *Malassezia pachydermatis*. *J. Mycol. Med.* 29 375–377. 10.1016/j.mycmed.2019.08.003 31455580

[B49] Shagdarova B. T. Ilyina A. V. Lopatin S. A. Varlamov V. P. Kartashov M. I. Arslanova L. R. (2018). Study of the protective activity of chitosan hydrolyzate against Septoria leaf blotch of wheat and brown spot of tobacco. *Appl. Biochem. Microbiol.* 54 71–75. 10.1134/S0003683818010118

[B50] Shcherbakova L. A. Nazarova T. A. Mikityuk O. D. Istomina E. A. Odintsova T. I. (2018). An extract purified from the mycelium of a tomato wilt-controlling strain of *Fusarium sambucinum* can protect wheat against Fusarium and common root rots. *Pathogens* 7:61. 10.3390/pathogens7030061 30011945 PMC6160971

[B51] Shcherbakova L. A. Syomina Y. V. Arslanova L. R. Nazarova T. A. Dzhavakhiya V. G. (2019). Metabolites secreted by a nonpathogenic *Fusarium sambucinum* inhabiting wheat rhizosphere enhance fungicidal effect of some triazoles against *Parastagonospora nodorum*. *AIP Conf. Proc.* 2063:030018. 10.1063/1.5087326

[B52] Shen Q. Zhou W. Li H. Hu L. Mo H. (2016). ROS involves the fungicidal actions of thymol against spores of *Aspergillus flavus* via the induction of nitric oxide. *PLoS One* 11:e0155647. 10.1371/journal.pone.0155647 27196096 PMC4872997

[B53] Stakheev A. A. Khairulina D. R. Zavriev S. K. (2016). Four-locus phylogeny of *Fusarium avenaceum* and related species and their species-specific identification based on partial phosphate permease gene sequences. *Int. J. Food Microbiol.* 225 27–37. 10.1016/j.ijfoodmicro.2016.02.012 26974249

[B54] Stakheev A. A. Samokhvalova L. V. Mikityuk O. D. Zavriev S. K. (2018). Phylogenetic analysis and molecular typing of trichothecene-producing *Fusarium* fungi from Russian collections. *Acta Naturae* 10 79–92. PMC6087817 30116619

[B55] State Catalogue of Pesticides and Agrochemicals (2018). *State Catalogue of Pesticides and Agrochemicals Approved For the Use in Russian Federation in 2020.* Moscow: Ministry of Agriculture of Russian Federation, 325.

[B56] Teixeira B. Marques A. Ramos C. Neng N. R. Nogueira J. M. F. Saraiva J. A. (2013). Chemical composition and antibacterial and antioxidant properties of commercial essential oils. *Ind. Crops Prod.* 43 587–595. 10.1016/j.indcrop.2012.07.069

[B57] Veras H. N. H. Rodrigues F. F. G. Botelho M. A. Menezes I. R. A. Coutinho H. D. M. Costa J. G. M. (2017). Enhancement of aminoglycosides and β-lactams antibiotic activity by essential oil of *Lippia sidoides* Cham. and the thymol. *Arab. J. Chem.* 10 S2790–S2795.

[B58] Villafana R. T. Ramdass A. C. Rampersar S. N. (2019). Selection of *Fusarium* trichothecene toxin genes for molecular detection depends on TRI gene cluster organization and gene function. *Toxins* 11:36. 10.3390/toxins11010036 30646506 PMC6357111

[B59] Zadoks J. C. Chang T. T. Konzak C. F. (1974). A decimal code for the growth of cereals. *Weed Res.* 14 415–421. 10.1111/j.1365-3180.1974.tb01084.x

[B60] Zhang Z. Qin G. Li B. Tian S. (2015). Effect of cinnamic acid for controlling gray mold on table grape and its possible mechanisms of action. *Curr. Microbiol.* 71 396–402. 10.1007/s00284-015-0863-1 26143055

